# Supplementary material for: USP14-regulated allostery of the human proteasome by time-resolved cryo-EM
Source: Nature. 2022 Apr 27;605(7910):567–74. doi: 10.1038/s41586-022-04671-8 (PMC9117149; doi:10.1038/s41586-022-04671-8)
Supplement: Supplementary file 1 — This file contains the raw (uncropped) gel images for Fig. 2 and for Extended Data Figs. 1, 9. [file 41586_2022_4671_MOESM1_ESM.pdf]

nature portfolio

https://doi.org/10.1038/s41586-022-04671-8

Supplementary information

USP14-regulated allostery of the human proteasome by time-resolved cryo-EM

In the format provided by the authors and unedited

Fig. 2h Anti-T7

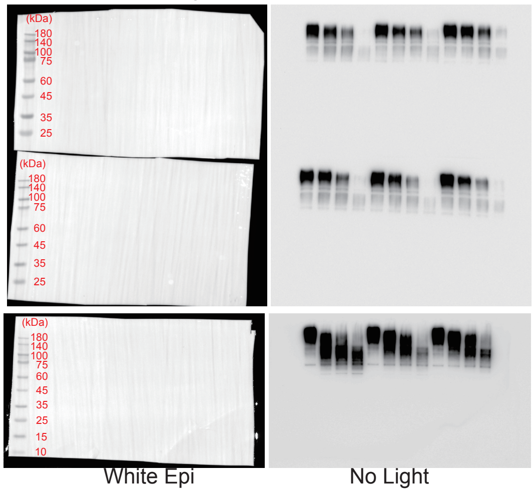

White Epi No Light

Extended Data Fig. 1b

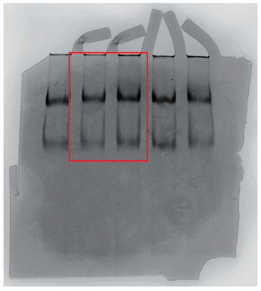

Extended Data Fig. 1d

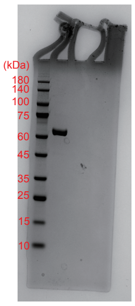

Extended Data Fig. 1e

RPN13

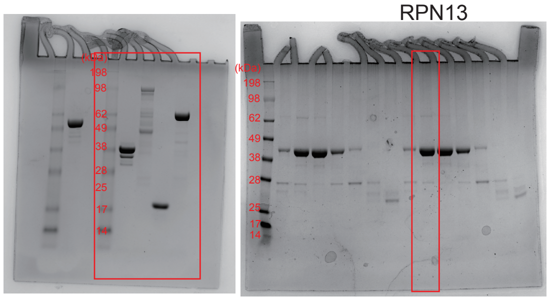

Extended Data Fig. 1f

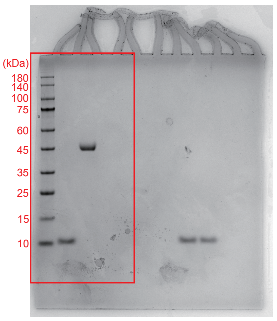

Extended Data Fig. 1g Anti-RPN13

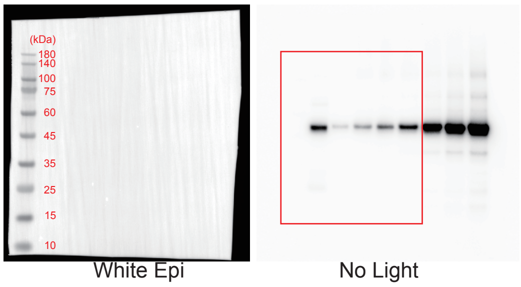

White Epi No Light

Extended Data Fig. 1h Anti-USP14

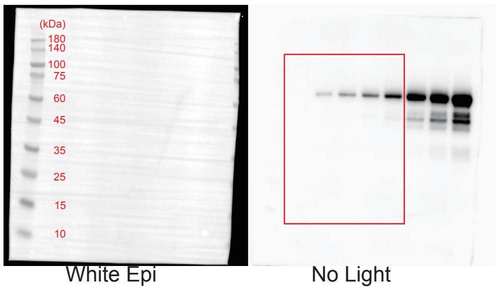

White Epi No Light

Extended Data Fig. 1i Anti-T7

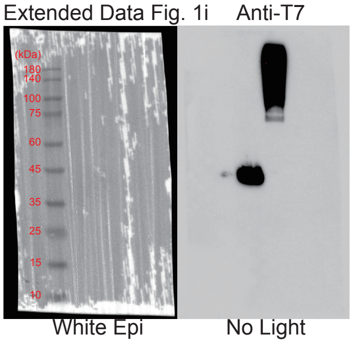

White Epi No Light

Extended Data Fig. 1j Anti-T7

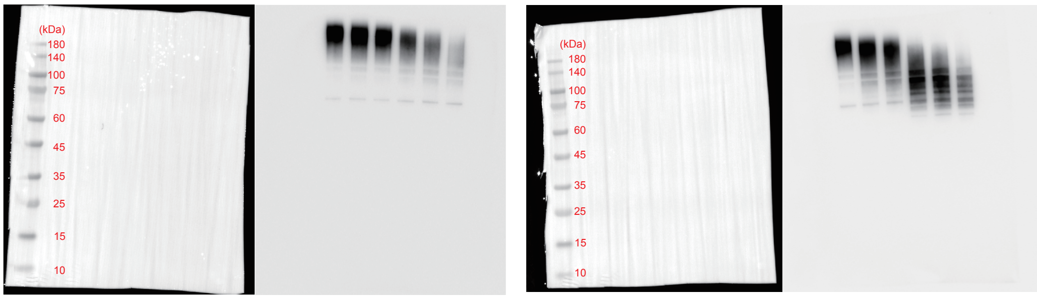

White Epi No Light White Epi No Light

Extended Data Fig. 9b

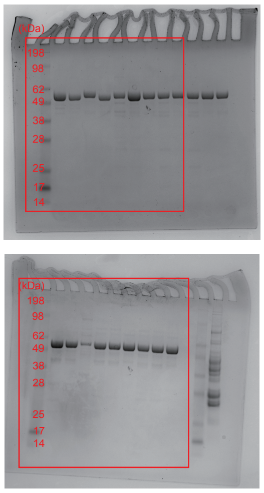

Extended Data Fig. 9g Anti-T7

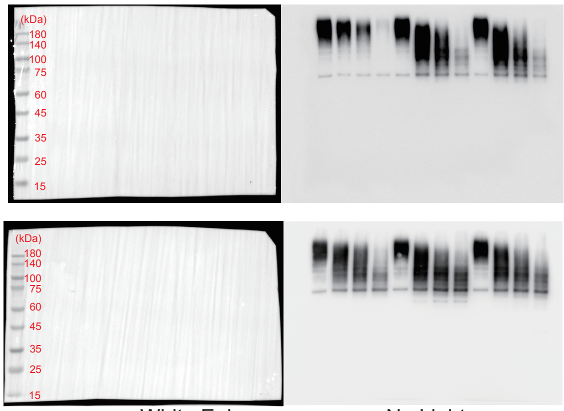

White Epi No Light

Extended Data Fig. 9h Anti-T7

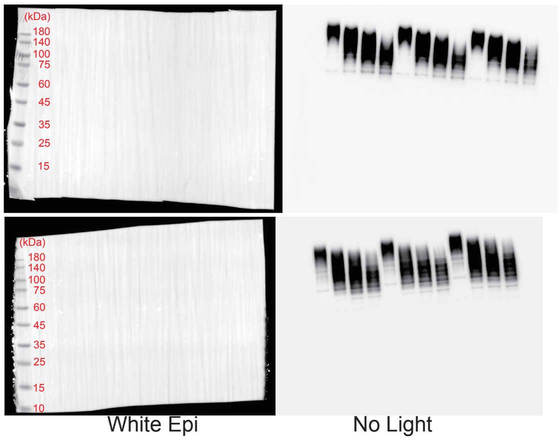

White Epi No Light
