# Supplementary material for: USP14-regulated allostery of the human proteasome by time-resolved cryo-EM
Source: Nature. 2022 Apr 27;605(7910):567–74. doi: 10.1038/s41586-022-04671-8 (PMC9117149; doi:10.1038/s41586-022-04671-8)
Supplement: Supplementary file 2 — Reporting Summary [file 41586_2022_4671_MOESM2_ESM.pdf]

nature portfolio

Corresponding author(s): Youdong Mao

Last updated by author(s): Feb 17, 2022

Reporting Summary

Nature Portfolio wishes to improve the reproducibility of the work that we publish. This form provides structure for consistency and transparency in reporting. For further information on Nature Portfolio policies, see our Editorial Policies and the Editorial Policy Checklist.

Statistics

For all statistical analyses, confirm that the following items are present in the figure legend, table legend, main text, or Methods section.

n/a	Confirmed	
☐	☒	The exact sample size (*n*) for each experimental group/condition, given as a discrete number and unit of measurement
☒	☐	A statement on whether measurements were taken from distinct samples or whether the same sample was measured repeatedly
☐	☒	The statistical test(s) used AND whether they are one- or two-sided<br>*Only common tests should be described solely by name; describe more complex techniques in the Methods section.*
☒	☐	A description of all covariates tested
☐	☒	A description of any assumptions or corrections, such as tests of normality and adjustment for multiple comparisons
☐	☒	A full description of the statistical parameters including central tendency (e.g. means) or other basic estimates (e.g. regression coefficient) AND variation (e.g. standard deviation) or associated estimates of uncertainty (e.g. confidence intervals)
☐	☒	For null hypothesis testing, the test statistic (e.g. *F*, *t*, *r*) with confidence intervals, effect sizes, degrees of freedom and *P* value noted<br>*Give P values as exact values whenever suitable.*
☒	☐	For Bayesian analysis, information on the choice of priors and Markov chain Monte Carlo settings
☐	☒	For hierarchical and complex designs, identification of the appropriate level for tests and full reporting of outcomes
☒	☐	Estimates of effect sizes (e.g. Cohen's *d*, Pearson's *r*), indicating how they were calculated

Our web collection on statistics for biologists contains articles on many of the points above.

Software and code

Policy information about availability of computer code

Data collection	SerialEM v3.6.11
Data analysis	MotionCor2 v1.2.1, GCTF v1.06, DeepEM v1.0, EMAN v2.22, SPIDER v22.10, ROME v1.1.2, RELION v3.1.3, AlphaCryo4D v0.1.0, COOT v0.9.5, Phenix v1.19.2, ResMap v1.1.4, Pymol v2.2.3, UCSF Chimera v1.16, ChimeraX v1.2.5, Image J v1.53, SPSS v27.0

For manuscripts utilizing custom algorithms or software that are central to the research but not yet described in published literature, software must be made available to editors and reviewers. We strongly encourage code deposition in a community repository (e.g. GitHub). See the Nature Portfolio guidelines for submitting code & software for further information.

Data

Policy information about availability of data

All manuscripts must include a data availability statement. This statement should provide the following information, where applicable:

- Accession codes, unique identifiers, or web links for publicly available datasets
- A description of any restrictions on data availability
- For clinical datasets or third party data, please ensure that the statement adheres to our policy

Cryo-EM density maps of USP14-proteasome complexes have been deposited in the Electron Microscopy Data Bank (EMDB) (www.emdatasource.org) under accession codes EMD-32272 (EA1UBL), EMD-32273 (EA2.0UBL), EMD-32274 (EA2.1UBL), EMD-32275 (ED4USP14), EMD-32276 (ED5USP14), EMD-32277 (ED0USP14), EMD-32278 (ED1USP14), EMD-32279 (ED2.0USP14), EMD-32280 (ED2.1USP14), EMD-32281 (SBUSP14), EMD-32282 (SCUSP14), EMD-32283 (SD4USP14) and EMD-32284 (SD5USP14), EMD-32285 (EA1UBL with the local RPN1 density improved), EMD-32286 (EA2.0UBL with the local RPN1 density improved), EMD-32287 (EA2.1UBL with the local RPN1 density improved), EMD-32288 (ED4USP14 with the USP14 density improved), EMD-32289 (ED0USP14 with the RPN1 density improved), EMD-32290 (ED2.0USP14 with the RPN1 density improved), EMD-32291 (ED2.1USP14 with the USP14 density improved), and

EMD-32292 (SCUSP14 with the USP14 density improved). The corresponding coordinates have been deposited in the Protein Data Bank (PDB) (www.wwpdb.org) under accession codes 7W37 (EA1UBL), 7W38 (EA2.0UBL), 7W39 (EA2.1UBL), 7W3A (ED4USP14), 7W3B (ED5USP14), 7W3C (ED0USP14), 7W3F (ED1USP14), 7W3G (ED2.0USP14), 7W3H (ED2.1USP14), 7W3I (SBUSP14), 7W3J (SCUSP14), 7W3K (SD4USP14) and 7W3M (SD5USP14). Comparisons to protein structures from previous publications used the atomic models in the PDB under accession codes: 2AYN (USP domain of USP14 in its isolated form), 2AYO (USP domain of USP14 bound to ubiquitin aldehyde), 6MSB (state EA1 of substrate-engaged human proteasome), 6MSD (state EA2), 6MSE (state EB), 6MSG (state EC1), 6MSJ (state ED1), 6MSK (state ED2), 5VFT (state SB of substrate-free human proteasome), 5VFU (state SC), 5VFP (state SD1), and 5VFR (state SD3). Cryo-EM maps from previous publications used in comparison are available from EMDB under access codes EMD-9511 (USP14-UbAl-bound proteasome), EMD-3537 (Ubp6-bound proteasome map) and EMD-2995 (Ubp6-UbVS-bound proteasome). Uncropped versions of all gels and blots are provided in Supplementary Fig. 1. All other data are available from the corresponding author upon reasonable request. Source data are provided with this paper.

Field-specific reporting

Please select the one below that is the best fit for your research. If you are not sure, read the appropriate sections before making your selection.

☒ Life sciences ☐ Behavioural & social sciences ☐ Ecological, evolutionary & environmental sciences

For a reference copy of the document with all sections, see nature.com/documents/nr-reporting-summary-flat.pdf

Life sciences study design

All studies must disclose on these points even when the disclosure is negative.

Sample size	No statistical methods were used to estimate appropriate sample size. The actual sample size was increased until the finally achieved resolution and quality of 3D reconstructions of the cryo-EM structures meet the expectation, that is, no worse than 3.6 Å by gold-standard Fourier shell correlation (FSC) measurement.
Data exclusions	No data were excluded from the analysis. During cryo-EM data clustering, good cryo-EM images were chosen for further 3D analysis based on their achieved resolution and reconstruction quality. Poorer images were excluded in the final reconstructions based on the criteria of maximizing the map resolution and quality.
Replication	All functional experiments were repeated at least three times. All attempts at replication were successful.
Randomization	Single-particle sets of cryo-EM images were randomly split for the purposes of estimating overall resolution during Fourier shell correlation calculation. Otherwise randomization was not relevant to these studies.
Blinding	Blinding was not relevant to this study.

Reporting for specific materials, systems and methods

We require information from authors about some types of materials, experimental systems and methods used in many studies. Here, indicate whether each material, system or method listed is relevant to your study. If you are not sure if a list item applies to your research, read the appropriate section before selecting a response.

Materials & experimental systems

n/a	Involved in the study	
☐	☒	Antibodies
☐	☒	Eukaryotic cell lines
☒	☐	Palaeontology and archaeology
☒	☐	Animals and other organisms
☒	☐	Human research participants
☒	☐	Clinical data
☒	☐	Dual use research of concern

Methods

n/a	Involved in the study	
☒	☐	ChIP-seq
☒	☐	Flow cytometry
☒	☐	MRI-based neuroimaging

Antibodies

Antibodies used	Anti-T7 (abcamAnti-T7 (abcam, cat#: ab9115, Rabbit polyconal, 1:1000)<br>Anti-RPN13 (abcam, cat#: ab157185, Rabbit monoclonal, 1;10000)<br>Anti-USP14(Novus, cat#: NBP2-20826, Rabbit polyconal, 1:1000)
Validation	Information of the antibody validation is available through manufacturer's online database. No further validation was done on the antibody in the reported experiments.

Eukaryotic cell lines

Policy information about cell lines

Cell line source(s)	A stable HEK293 cell line harboring HTBH (hexahistidine, TEV cleavage site, biotin, and hexahistidine) tagged hRPN11 , a gift from L. Huang, Departments of Physiology and Biophysics and of Developmental and Cell Biology, University of California, Irvine, California 92697.
Authentication	Further authentication was not performed for this study.
Mycoplasma contamination	Mycoplasma testing was not performed for this study.
Commonly misidentified lines (See ICLAC register)	No commonly misidentified cell lines were used in this study.
